# Genome Sequence of a *Minacovirus* Strain from a Farmed Mink in The Netherlands

**DOI:** 10.1128/MRA.01451-20

**Published:** 2021-02-25

**Authors:** Kirsty T. T. Kwok, Myrna M. T. de Rooij, Felisita F. Sinartio, Lidwien A. M. Smit, Marion P. G. Koopmans, My V. T. Phan

**Affiliations:** a Department of Viroscience, Erasmus MC, Rotterdam, The Netherlands; b Institute for Risk Assessment Sciences (IRAS), Utrecht University, Utrecht, The Netherlands; KU Leuven

## Abstract

We report the genome sequence of a *Minacovirus* strain identified from a fecal sample from a farmed mink (Neovison vison) in The Netherlands that was tested negative for severe acute respiratory syndrome coronavirus 2 (SARS-CoV-2) using real-time PCR (RT-PCR). The viral genome sequence was obtained using agnostic deep sequencing.

## ANNOUNCEMENT

The subgenus *Minacovirus* belongs to the genus *Alphacoronavirus*, a member of the *Coronaviridae* family of viruses with a linear positive-sense single-stranded RNA genome. Alphacoronaviruses have been found in multiple mammals, including humans, bats, minks, ferrets, pigs, cats, and dogs, and can cause respiratory and gastrointestinal illnesses (1). *Minacovirus* strains have been identified in minks and ferrets and are potentially associated with epizootic catarrhal gastroenteritis in both animals (2, 3).

Here, we report the genome sequence of the *Minacovirus* strain Mink/Minacovirus/NLD/2020/NT_4, identified from a fecal sample collected from a farmed mink. Fecal samples from minks (Neovison vison) were collected on mink farms with reported severe acute respiratory syndrome coronavirus 2 (SARS-CoV-2) infections in The Netherlands in May 2020 as part of the outbreak investigation (4, 5). Twelve samples that tested negative for SARS-CoV-2 using real-time PCR (RT-PCR) (6) were subjected to metagenomic sequencing as part of a virome study. A coding-complete *Minacovirus* genome sequence was obtained from one sample collected in North Brabant Province. A fecal suspension (30% [wt/vol]) was prepared in phosphate-buffered saline and then subjected to centrifugation (10,000 × *g*, 10 min) and DNase treatment (37°C, 30 min) (TURBO DNase; Invitrogen). Nucleic acid was extracted using a QIAamp viral RNA minikit without adding any carrier RNA and then reverse transcribed using SuperScript III reverse transcriptase (Invitrogen) and nonribosomal random hexamers (7). Second-strand cDNA was synthesized using Klenow fragment (3′ to 5′ exonuclease deficient; NEB). A library was prepared using the Nextera XT DNA library prep kit (Illumina) and then subjected to paired-end sequencing on the MiSeq platform (600-cycle reagent kit v3; Illumina).

A total of 1,995,588 paired-end reads were generated. Paired fastq files were analyzed using the Web-based automatic Genome Detective v1.126 pan-viral typing tool (8). In brief, Trimmomatic (9) was used for removing adapters and quality trimming, followed by viral read identification using DIAMOND (10). The sorted viral reads were *de novo* assembled using metaSPAdes (11). The *de novo* assembled genome sequence was examined using Geneious v2020.2.3 (12) and annotated using VAPiD (13). All tools were used with default parameters unless otherwise specified. The coding-complete *Minacovirus* genome sequence is 28,868 nucleotides long with a depth of coverage of 1,031×, 218,966 mapped reads, and a GC content of 37.6%. A nucleotide BLAST search showed that this strain shares 90.5% to 91.8% similarity at the nucleotide level with the mink coronavirus 1 strains MCoV1/11917-2/DK/2015 and MCoV1/11918-1/DK/2015 from Denmark (GenBank accession numbers MN535736 and MN535737), mink coronavirus strains WD1127 and WD1133 from the United States (HM245925 and HM245926), and *Alphacoronavirus* strain Mink/China/1/2016 from China (MF113046). In a comparison of the spike protein amino acid sequences using a protein BLAST search, the reported genome sequence Mink/Minacovirus/NLD/2020/NT_4 shared 87.4% to 92.5% similarity with the abovementioned strains from Denmark, the United States, and China.

In conclusion, we report a *Minacovirus* genome sequence identified from a farmed mink in The Netherlands. Follow-up surveillance is warranted to investigate the prevalence and clinical implications of the virus. Understanding coronavirus diversity in minks is vital for both mink disease and zoonosis preparedness, given that minks have shown the ability to harbor coronaviruses, including SARS-CoV-2 (4), and mink farming has been heavily impacted by ongoing SARS-CoV-2 outbreaks on the farms (14, 15).

### Data availability.

The genome sequence described in this study (Mink/Minacovirus/NLD/2020/NT_4) has been deposited in GenBank under accession number MW248736. The short reads have been deposited in SRA under accession number SRX9605666 (BioProject accession number PRJNA681552 and BioSample accession number SAMN16956096).
